# Transmission of Hundred-keV Protons through Insulating Nanocapillaries: Charge-patch-assisted Specular Reflections

**DOI:** 10.1038/srep15169

**Published:** 2015-10-15

**Authors:** G. Y. Wang, J. X. Shao, Q. Song, D. Mo, A. X. Yang, X. Ma, W. Zhou, Y. Cui, Y. Li, Z. L. Liu, X. M. Chen

**Affiliations:** 1 School of Nuclear Science and Technology of Lanzhou University, Lanzhou, 730000, China; 2 Institute of Modern Physics, Chinese Academy of Sciences, Lanzhou, 730000, China

## Abstract

In this work, we measured the time evolution of the transmission features of 10–100 keV protons transmitted through nanocapillaries in a polycarbonate (PC) membrane. After reaching equilibrium, transmitted particles with an incident energy of 100 keV were located around the direction along the incident beam but not along the capillary axis, indicating that the transport mechanism of the 100 keV ion was distinct from that of keV-energy ions. The simulation results indicated that charge-patch-assisted collective scatterings on the surface are the main transport mechanism for the hundred-keV ions in nanocapillaries. This scenario fills in the gap in the previous understanding of ion transmission in nanocapillaries from keV to MeV energies.

Nanocapillaries in membranes have been widely applied in the areas of physics, chemistry, biology and materials science. These membranes are used as masks for ion implantations^1^, switchable ion-transport gates^2^,^3^, filters for biological molecules^4^,^5^, and sensors to transport micro-sized ion beams^6^,^7^,.

N. Stolterfoht *et al.* recently discovered a remarkable guiding effect for keV-energy ions transmitted through insulating nanocapillaries^8^,^9^,^10^. After a charge-up phase, the incident ions entering the insulating nanocapillaries can be effectively steered along the tilted capillary axis. This effect results from self-organized charge patches^11^,^12^,^13^ on the capillary walls that subsequently deflect the ions electrostatically, which inhibits close collisions with the capillary walls. This type of guiding process has also been observed in experiments with slow negative ions transmitted through insulating nanocapillaries^14^.

Simulations of electron transport through capillaries^15^ have shown that the mechanism for guiding electrons is fundamentally different from that for guiding ions. The stochastic sequence of elastic and inelastic scatterings below the internal capillary surface, rather than the self-organized charge patch, is the key to electron transmission along the capillary axis.

Measurements and simulations of the steering of swift ions at MeV energies have shown that the transmission of MeV ions^16^,^17^ is similar to that of moderately energetic electrons (less than 1 keV)^18^. The charges deposited on the capillary walls do not affect the ion transmission, whereas multiple stochastic scatterings below the surface lead to the phenomenon of guided transmission of MeV ions. A trajectory simulation using Rutherford scattering in close projectile-target ion binary encounters succeeded in qualitatively reproducing the shape and energy distribution of the transmitted ions^19^.

Because the energy loss and irradiation effect of hundred-keV ions on the surface are considerably stronger than those of keV and MeV ions, microsized or nanosized ion beams with hundred-keV energies have broad applications, ranging from microsurgery in living cells^20^,^21^,^22^ to the controlled nanopatterning^23^,^24^,^25^ of material surfaces, e.g., forming regular nanopore arrays on graphene^26^,^27^.

The key to applying a hundred-keV nanosized ion beam formed by nanocapillaries is to understand the transmission features of the ions inside the capillaries. To date, few experimental or theoretical investigations have been conducted for hundred-keV energies. What is the transmission mechanism of hundred-keV ions in an insulating nanocapillary, and is it special and distinct from those of keV and MeV ions? These are important and unanswered questions.

In this work, we measured the time evolution of the transmission features, such as the charge states and position distributions, and the relative transmission rate of 10–100 keV protons transmitted through nanocapillaries in a PC membrane at a tilt angle of +1°. The experimental data clearly show that the transmission mechanism of 100 keV protons is distinct from that of 10 keV protons. After a sufficient charge-up period, 10 keV projectiles are guided through the capillaries, and the transmitted particles are located around the guiding direction of the capillary axis. However, for 100 keV projectiles, the centroid outgoing angle of the transmitted particles gradually shifts from the guiding direction to the direction of the incident beam during the later measurement stage; the centroid outgoing angle of the transmitted particles remains in this direction.

## Experiments

**Figure 1:**
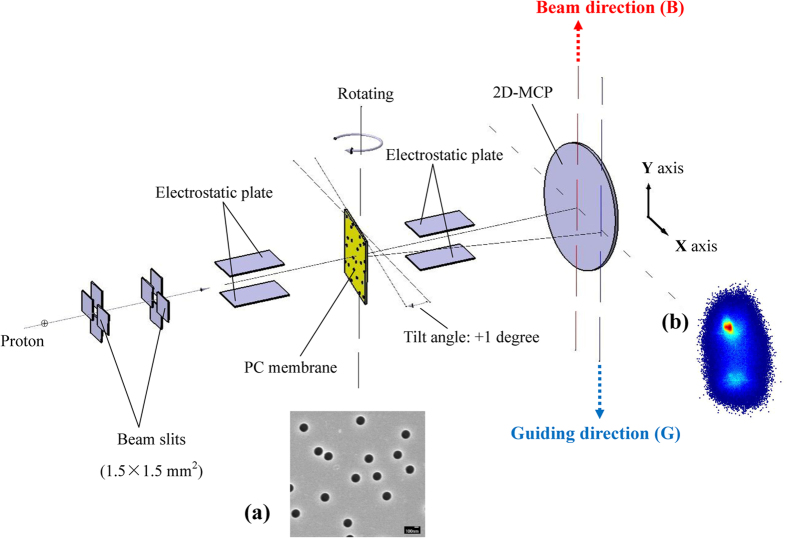
Schematic diagram of the experiment. Inset (**a**): SEM image of the PC nanocapillary membrane. Inset (**b**): the typical 2D spectrum of transmitted particles with 100-keV incident energy and with a tilt angle of +1°.

A schematic diagram of the experiment is shown in Fig. 1. Proton beams with energies of 10–100 keV are extracted from the 14.5 GHz ECR ion source at the Institute of Modern Physics, Chinese Academy of Sciences. After being collimated by two sets of 1.5 × 1.5 mm^2^ beam slits separated by a distance of 75 cm, the beam collides with the PC membrane at an intensity of 1 nA/mm^2^ and a divergence of 0.2°. The PC membrane with nanocapillaries is hung at the center of a high-vacuum chamber at a pressure of 10^−6^ Pa. The tilt angle between the incident beam direction and the normal direction of the membrane is determined by rotating the target around the vertical axis with an accuracy of 0.1°. The azimuthal angle is adjusted using a pair of electrostatic plates located 10 cm before the target. The transmitted particles with different charges are separated by another pair of electrostatic deflection plates located 7 cm behind the target. Using the electrostatic plates, transmitted protons and hydrogen atoms are separated along the *y*-axis, and distortion of the position distributions along the *x*-axis is avoided. The position distributions of the transmitted particles are recorded using a two-dimensional position-sensitive (2D-MCP) detector, which is located 30 cm behind the target. Before the measurement, the “zero position”, which is where the capillaries inside the membrane are fully parallel to the incident beam direction, had to be established. We carefully adjust the tilt angle and azimuthal angle to obtain the maximum counting rates on the 2D-MCP detector, and then, the “zero position” is set.

The 30-μm-thick PC membranes were previously irradiated by 11.4 MeV/u U ions at the UNILAC linear accelerator at GSI. Given their large mass and high energy, the projectiles pass through the entire thickness of the foils and produce tracks that are a few nanometers in diameter. The irradiated membranes are then etched in a 5 M NaOH solution at 50 °C to change the latent tracks into nanocapillaries. The capillaries have a diameter of 200 nm and a length of 30 μm (aspect ratio of 150:1, corresponding to an angular opening of 0.38°). Inset (a) in Fig. 1 shows an SEM image of the membranes, and we verified that the openings of the capillaries are circular. The nanowires fabricated within these capillaries indicate that the inner walls of the nanocapillaries have excellent cylindrical shapes with smooth and homogeneous contours^28^. To avoid charging up of the front surface, both surfaces of the membrane were evaporated with Au at 45° with a thickness of 30 nm.

Inset (b) of Fig. 1 shows the typical 2D-MCP spectrum of transmitted particles after the 100 keV protons pass through the PC nanocapillaries with a tilt angle of +1°, as measured in the later measurement stage. The upper and lower charge patches show the transmitted protons and hydrogen atoms, respectively. These two patches are isolated and independent from each other, and the background count is small.

## Results

**Figure 2:**
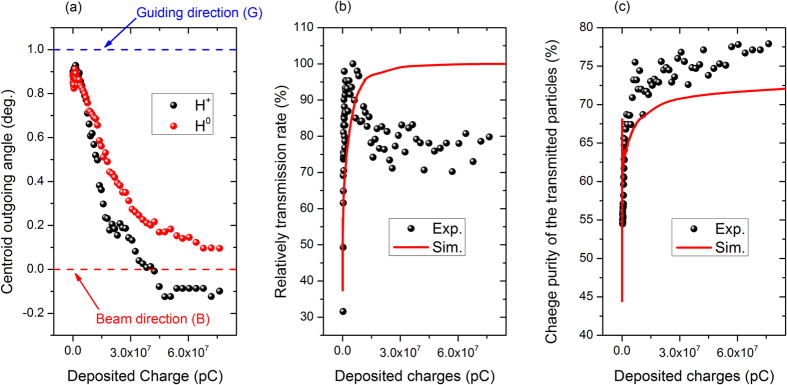
Evolution of the (a) centroid angle, (b) relative transmission rate, and (c) charge purity of transmitted particles at an incident energy of 100 keV and a membrane tilt angle of +1°.

Figure 2(a) shows the time evolution of the centroid angle of the transmitted particles at an incident energy of 100 keV and a tilt angle of +1°. The abscissa in Fig. 2(a) is the total charge of the incident protons received by the front surface of the PC membrane. As shown in Fig. 2(a), at the beginning of the measurement, the centroid angles of the transmitted protons and hydrogen atoms are located in the vicinity of +0.9°, which indicates that the particles are transmitted along the capillary axis. With the charge-up, the centroid angle of the transmitted particles gradually shifts to the beam direction and then remains in the range of 0° ± 0.2°

Fig. 2(b) shows the variation of the relative transmission rate of the transmitted particles with the amount of charge deposited on the front surface of the membrane. In the early measurement stage, the particle transmission rate increases rapidly to its maximum value and then declines gradually. Finally, the transmission rate remains within the range of 70–80%. Figure 2(b) illustrates that the charges deposited in the capillaries will affect the transmission rate. The slow decline of the transmission rate after it achieves its peak is caused by the blocking effect^29^. Figure 2(c) shows the evolution of the charge purity of the transmitted particles over time. The relative proportion of protons in the transmitted particles initially increases rapidly from 54% to 70%, which is similar to the rapid increase in the transmission rate. The charge purity then increases slightly and remains within the range of 75–80%. Figure 2 indicates that the charges deposited in the capillaries can increase the transmission rate and outgoing charge purity of 100 keV incident protons. After charge and discharge equilibrium, the projectiles are stably transmitted along the direction of the incident beam but not along the capillary axis, as in a keV-energy beam.

**Figure 3:**
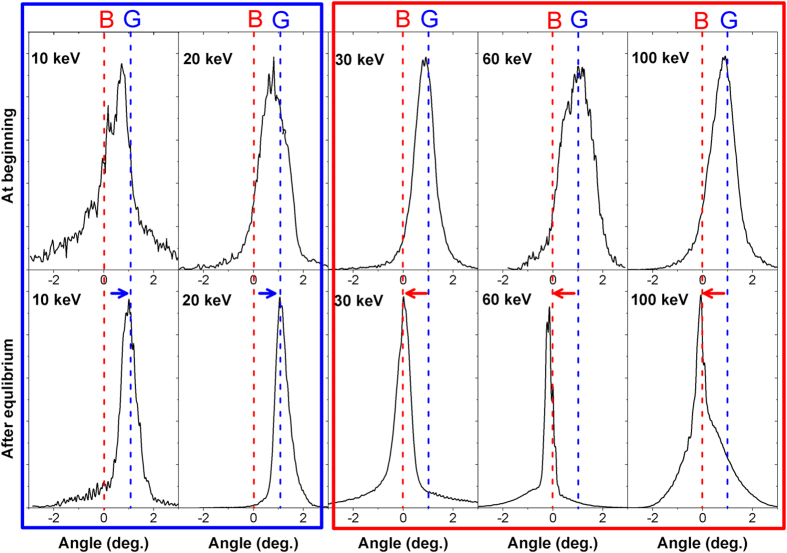
Measured angular distribution of the transmitted particles with proton incident energies of 10–100 keV and a 1° tilt angle for the PC membrane. The upper row shows spectra obtained at the beginning of the measurement, and the lower row show spectra obtained after equilibrium.

To show the distinct transmission features for projectiles with different incident energies, the angular distribution spectra of transmitted particles with incident energies from 10 to 100 keV measured at the early and later stages of measurement are plotted in Fig. 3. In the left part (the blue box) of Fig. 3, at the beginning of the measurement, transmitted particles with incident energies of 10 and 20 keV are located around the centroid angle of +0.6°, and the FWHMs are approximately 1–1.2°. After charge and discharge equilibrium is achieved, the centroid angles of the transmitted particles shift from left to right (the blue arrow) to 1° and are along the guiding direction (G line) of the capillary axis. The spectra are near-Gaussian distributions, and the FWHMs decrease to 0.6–0.7°.

The right part (the red box) of Fig. 3 illustrates that the angular distributions of the transmitted particles with 30–100 keV incident energies are quite different from those at 10 and 20 keV. Transmitted particles are initially nearly symmetrically centered at angles of approximately 0.9–1.1°, and their FWHMs are 1.2–1.4°. After equilibrium is achieved, both the centroid angle and spectrum shape change significantly. The centroid angles move from right to left (the red arrow) to approximately 0° and are no longer along the guiding direction but instead along the incident beam direction (B line). The shape of the spectra changes from a nearly Gaussian distribution to a sharp narrow peak on a low broad shoulder, and the FWHMs are only 0.3–0.5°.

These results illustrate that the dynamic guiding process is dominant at low energies of approximately 10 keV. For higher energies near 100 keV, although the deposited charge patches play a role in the transmission, the transmission of intermediate-energy ions in nanocapillaries is unique and considerably different from that at lower energies.

### Simulation

The simulation was conducted to understand the underlying scenario of hundred-keV ion transmission through insulating nanocapillaries and to identify the primary reason for the distinct transmission mechanisms of ions with different energies ranging from keV to hundreds of keV and even MeV.

Regardless of the projectile’s energy, three different types of forces will be exerted on the projectile when it is inside the capillary. The first type of force is the long-range Coulomb force from the deposited surface charges on the inner capillary wall. The second is the short-range collective scattering force from the topmost surface layer atoms. The third is the binary encounter force between the projectile and target atoms below the internal surface. When the projectile is above the surface, the Coulomb force and the collective surface scattering force work simultaneously. And the stochastic binary encounter force does not exist because the projectile does not penetrate into the inner capillary surface yet. But when the projectile penetrates into the surface, the binary encounter force is considered. That is, all of these three kinds of forces are taken into account when the projectile is under the surface. The importance of these three types of forces will be reversed with charge-up. After equilibrium, the greatest force will determine the ions’ transmission features in different energy ranges. In this context, we will introduce the detailed simulation methods used to describe these three forces and the projectile’s motion inside the capillary.

#### A. Above the surface: Coulomb force from the deposited charges

With the approach of a projectile, when the vertical distance from the incident proton to the inner capillary wall is less than the charge-exchange distance 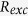, the proton will capture an electron, and a positive charge will be deposited onto the surface. The charge-exchange cross section between the 100 keV proton and an H_2_O molecule is 4.8 × 10^−17^ cm^2^ 30, and the cross section for an H_2_ molecule is 2.1 × 10^−17^ cm^2^ 31. According to Bragg’s Law, the average charge-exchange cross section between the 100 keV proton and the atoms in the C_16_H_14_O_3_ molecule is 1.9 × 10^−17^ cm^2^. Therefore, the vertical charge-exchange distance of the 100 keV proton from the PC surface is 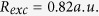


After a proton captures an electron and becomes a hydrogen atom, the hydrogen atom has a probability of losing an electron and turning into a proton again in the subsequent collisions. The vertical distance at which the electron loss will occur is 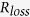, and the calculation method of 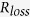 will be provided in Subsection (C).

Charges deposited on the surface will diffuse and attenuate gradually. The time evolution of the deposited charges follows the equation
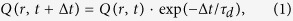
where 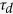 is the surface discharge time constant of the PC membrane. In this paper, 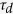 is 5 min. In a simulation of the guiding effect of the PET membrane, K. Schiessl set the discharge time of the PET membrane to 2–5 min^32^, and the resulting calculation results were consistent with the experimental data. Considering that the conductivity of the PC membrane is highly similar to that of the PET membrane, the definition of 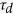 (several minutes) in this paper is reasonable^33^.

As surface charge patches are formed, the subsequent incident protons will experience long-range Coulomb forces from the surface charges. Here, 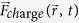 is the total Coulomb force between the charges deposited on the inner capillary wall and the incident proton at position 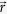 and time *t*.

#### B. Near the surface: surface layer atomic collective scattering

In this work, the incidence angle of the 100 keV proton is small; thus, the proton and hydrogen atom may experience the collective scattering forces from a larger number of the topmost surface layer atoms when they are sufficiently close to the surface. The scattering potential between a surface atom and proton (hydrogen atom) is the well-known Moliere potential:
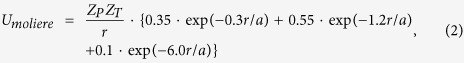
where 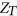 is the nuclear charge number of each surface atom and 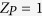 for protons or a hydrogen atom.

In the calculation, the surface is defined as unsmooth, and the surface layer atoms are randomly arranged around an average atom spacing 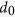, which is obtained from the equation
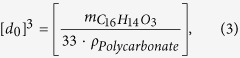
where 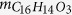 is the mass of a C_16_H_14_O_3_ molecule, 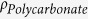 is the mass density of the PC membrane and 33 is the number of atoms in one C_16_H_14_O_3_ molecule. In the random arrangement, the occurrence probabilities of C, H, and O atoms in the PC membrane are determined by the molecular formula C_16_H_14_O_3_ and are 48.5%, 42.4%, and 9.1%, respectively. We name the collective scattering force between the surface layer’s atoms and the incident projectile 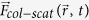; this force is the sum of the short-range scattering forces between all surface atoms and the projectile at position 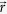 and time *t*.

#### C. Under the surface: stochastic binary encounters

When the projectile penetrates into the inner capillary surface, it will be surrounded by many target atoms; therefore, a series of stochastic binary encounters with single target atoms will occur. For simplicity and to reduce the calculation time considerably, the Coulomb potential between two bare nuclei is used to calculate the scattering orbit. Considering the assumptions made above, the differential scattering cross section is expressed by the well-known Rutherford formula:
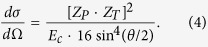
After integrating the differential scattering cross sections, we obtain the total scattering cross section 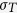, and the mean free path *λ* between two individual collisions is
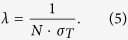
The probability *p*(*l*) that the projectile has a collision between *l* and *l* + *dl* follows the Poisson distribution
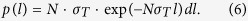
For the projectile energy considered here (100 keV), the projectile is swift, and its outer-shell electron will be lost or re-captured during collisions. The average charge state 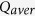 of the projectile is estimated by N. Bohr’s equation:
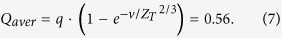
That is, approximately 56% of the projectiles are protons and 44% are hydrogen atoms when they are moving under the surface.

Therefore, the relationship between the electron-loss cross section 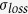 and charge-exchange cross section 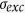 satisfies the following equation:
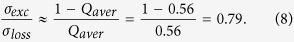
Then, we obtain 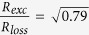, and 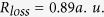


Several random numbers are generated to reproduce the physical distributions of the scattering angles, collision probabilities and charge states of the projectiles when they are under the surface.

During the flight between two elastic collisions, projectiles are decelerated through inelastic processes via the ionization or excitation of the target atoms. An analysis of our simulation data indicated that projectiles undergo binary encounters below the surface only two or three times before they re-escape from the surface. Therefore, the projectile energy loss via inelastic collisions is neglected.

#### D. Projectile trajectories

Before the projectile penetrates the surface, the force applied to the projectile is the sum of the long-range Coulomb force from the surface charge patches and the short-range collective scattering force from the surface layer’s atoms. Therefore, the total Hamiltonian can be expressed as
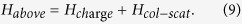
The long-range Coulomb force of the surface charge patches primarily works at a relatively far distance, whereas the hard, short-range collective scattering force of the surface layer’s atoms is important in the vicinity of the surface.

The generalized coordinates and momenta of the projectile during the transmission through the capillaries are obtained by solving Hamilton’s equation of motion:
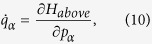

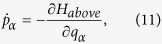
where 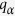 and 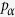 are the generalized coordinate and momentum of the projectile, respectively. We solve the Hamilton equation with the Dormand-Prince numerical algorithm to obtain the ion’s trajectory and ensure the calculation’s accuracy. For the motion of the projectile below the surface, we simulated the trajectory, scattering angle and charge state distribution during binary encounters, as noted in Subsection (C), using the Monte Carlo method.

## Discussion

**Figure 4:**
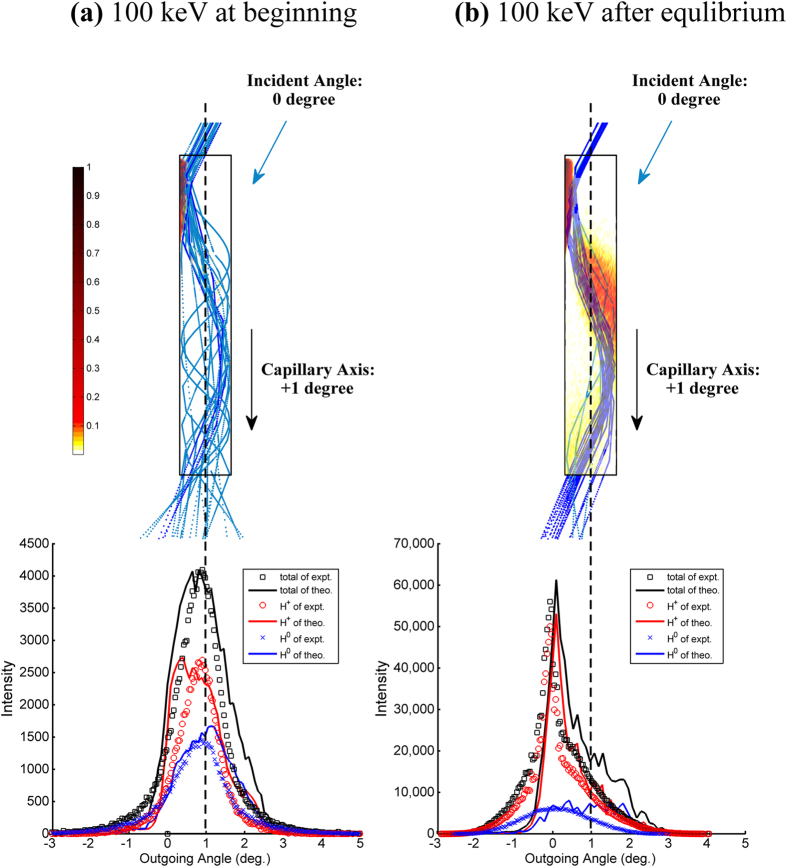
(Lower) Angular distribution of the transmitted particles after 100-keV protons pass through the nanocapillaries with a tilt angle of +1° (a) at the beginning and (b) after equilibrium. (Upper) Simulated drawings of charge patches and trajectories (**a**) at the beginning and (**b**) after equilibrium is achieved.

Figure 4(a,b) show a comparison between the experimental data and theoretical results for the angular distribution of the transmitted particles after 100 keV protons are transmitted through PC nanocapillaries in the early measurement stage and after charge-discharge equilibrium, respectively. The comparison indicates that the theoretical model can provide accurate descriptions of the angular distribution of the transmitted particles and the charge state in the early and later measurement stages.

As shown in Fig. 4(a), in the early measurement stage, the centroid angle of the transmitted protons and hydrogen atoms are in the vicinity of +1° and follow an approximately symmetrical Gaussian distribution. This feature is well described by the simulation. The measured charge purity is 54.5%, which is consistent with the theoretical value of 59.6%. The FWHM of the angular distribution spectrum of the transmitted particles is 1.32°, which is largely consistent with the theoretical value of 1.68°. In the simulation, nearly no charge patch is formed inside the capillaries in the early measurement stage, and most of the incident 100 keV protons will penetrate the inner capillary surface. Multiple random binary encounters will make the ions’ trajectories tortuous in the capillary, as shown in Fig. 4(a). As a result, particles are eventually transmitted along the direction of the capillary axis. Moreover, irregular random collisions significantly increase the FWHM of the angular distribution spectrum of the transmitted particles such that the FWHM is considerably larger than the geometric opening angle of the capillaries (0.38°). In addition, the average charge purity of the transmitted particles corresponds with the average charge equation for the passage of ions through matter^16^. In short, in the absence of charge deposition at the beginning of the measurement, the main transport mechanism of 100 keV protons is the multiple-scattering process via stochastic binary encounters below the surface.

As shown in Fig. 4(b), after charge-discharge equilibrium is achieved, the angular distribution of the transmitted particles is significantly different from its initial shape, as shown in Fig. 4(a). The centroid angle of the transmitted particles obtained via experiments and the theoretical model are approximately +0°. The spectrum shape is also significantly changed from the initial Gaussian distribution to an asymmetric distribution with a sharp peak on the left and a gentle shoulder on the right. The FWHM of the spectrum is 0.33°, which is consistent with the theoretical value of 0.51°. Moreover, the charge distribution of the transmitted particles obtained using the theoretical model is also reasonable: more than 72% of the transmitted ions still maintain their initial charge states.

For a 100 keV incident proton, the simulation shows that the surface charge patches cannot overcome all of the projectile’s vertical energy at large distances from the surface, but they still have a large probability of inhibiting the projectile from entering the surface. Therefore, the large majority of the projectiles can approach a distance close to the inner capillary wall but cannot penetrate the wall.

When the projectile is approaching the near surface, it will experience strong collective scattering forces from the surface layer’s atoms and undergo a large number of small angle scatterings, which lead to specular reflection from the topmost atomic layers and resemble surface channeling. After experiencing another specular reflection at a charge patch on the opposite side of the second half-capillary wall, the proton will be transmitted through the capillary along the direction of the incident beam. Because this type of transport mechanism is similar to two specular reflections, the FWHM of the angular distribution of the transmitted particles is constrained to be narrow by the geometric opening angle of the capillaries.

After the charge patches become stable, a small portion of the incident protons can still enter the charge-exchange distance 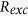 and become hydrogen atoms. These hydrogen atoms are not significantly affected by the charge patches and may penetrate the surface. Then, these hydrogen atoms lose memory of their charges during random collisions below the surface and are eventually transmitted through the capillary. Therefore, the FWHM of the angular distribution of the transmitted hydrogen atoms is 2.1°, which is largely consistent with the theoretical value of 2.5°. This feature of hydrogen atoms is different from that of transmitted protons.

The simulation results for the relative transmission rate and charge purity of the transmitted particles are shown in Fig. 2(b,c), respectively. The simulation can produce the main trends obtained in the experiment. More particles can be transmitted through the capillaries with the growth of the surface charge patches and under the specular scattering role of the near surface; the transmission rate will increase significantly as a result. Meanwhile, the Coulomb force of the charge patches will prevent incident protons from entering the charge-exchange distance, thus significantly increasing the charge purity; this trend is also observed.

**Figure 5:**
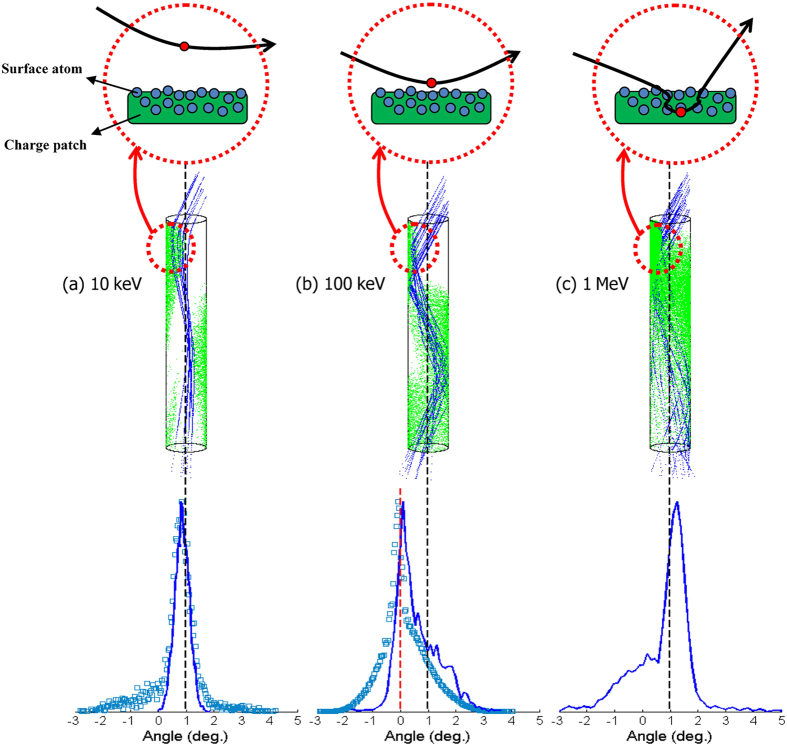
Measured data (dots) and simulation results (solid line) of the angular distribution spectrum of the transmitted particles after equilibrium for protons with incident energies of (a) 10 keV, (b) 100 keV and (c) 1 MeV and a tilt angle of the PC membrane of +1°.

The measured and simulated angular distributions of the projectiles with incident energies of 10 keV, 100 keV and 1 MeV transmitted through the PC nanocapillaries after the charge-discharge equilibrium are shown in Fig. 5, which illustrates that their transmission features are all quite distinct from each other.

Figure 5(a) illustrates that 10 keV projectiles are transmitted through the capillaries after charge-discharge equilibrium has been achieved. After the projectiles have collided with the first deposited charge patch near the entrance, the transverse momentum of the incident 10 keV protons is diminished by the charge patch’s repulsive force, and the protons’ trajectories are nearly parallel to the capillary axis. After a minor adjustment by the second and third charge patches, the particles will be transmitted along the capillary axis.

In contrast, in Fig. 5(c), the transverse momentum of the incident 1-MeV proton is too large, and the surface charge patches cannot play any role in the transmission. In the simulation, no time evolution is found for the transmission features of 1-MeV protons. All of the protons are transmitted through the capillary via multiple stochastic binary encounters below the surface. This type of stochastic binary encounter makes the trajectories of 1-MeV protons zigzag and appear irregular inside the capillary. The proton in a zigzag flight only exit along the capillary axis, and “phenomenon guiding” is observed.

Figure 5(b) illustrates that the transmission features of the 100 keV protons are significantly different than the features of the 10 keV and 1 MeV protons after equilibrium is reached. For the 100 keV protons, with the assistance of the guiding force of the surface charge patches, the collective scattering forces from the surface layer’s atoms will make the projectile’s trajectory look similar to mirror reflections. After two mirror reflections, the transmitted particles are centered in a direction close to the incident beam, which is not along the capillary axis. The transmission mechanism of the hundred-keV ions through the insulating nanocapillary is charge-patch-assisted specular reflection or the nanoscale surface channeling effect.

For low energies of several keV, the guiding force from the deposited surface charge patches is dominant. For high energies of MeV, the binary encounter force below surface is the most important. For intermediate energies of hundreds of keV, the charge-patch-assisted collective scattering force on the surface is crucial.

## Conclusion

We measured the time evolution of the angular distribution, relative transmission rate, and charge state of particles transmitted through PC nanocapillary membranes for incident proton energies of 10 to 100 keV. The transport mechanism of 100 keV ions was significantly different from that of keV-energy ions. We also simulated the major transmission features of ions in nanocapillaries with incident energies ranging from keV to hundreds of keV and MeV; good agreement with the experimental data was observed. A scenario was proposed in which the charge-patch-assisted surface channeling effect is the primary transport mechanism for hundred-keV ions transmitted through the insulating nanocapillary. This work also provides a more complete understanding of the passage of ions through nanocapillaries in broad energy ranges from keV to MeV.

## Additional Information

**How to cite this article**: Wang, G. Y. *et al.* Transmission of Hundred-keV Protons through Insulating Nanocapillaries: Charge-patch-assisted Specular Reflections. *Sci. Rep.* **5**, 15169; doi: 10.1038/srep15169 (2015).
